# On Simulated, or Feigned Diseases

**Published:** 1825-08

**Authors:** A. Copland Hutchison


THE LONDON
### Medical and Physical Journal.

No. 2 OF VOL. LIV.] AUGUST, 1825. [No. 318.

For many fortunate discoveries in medicine, and for the detection of numerous errors, the world is indebted to the rapid circulation of Monthly Journals; and there never existed any work, to which the Faculty, in Europe and America, were under deeper obligations, than to the Medical and Physical Journal of London, now forming a long, but an invaluable, series.—RUSH.

### ORIGINAL COMMUNICATIONS,
### SELECT OBSERVATIONS, &c.

ART. I.—*On Simulated, or Feigned Diseases.* By A. COPLAND HUTCHISON, Esq. &c. &c. &c.

[Continued from Vol. li. of this Journal.]

IN my former hastily drawn-up paper on Feigned Diseases, and inserted in your Number for February 1824, I find myself in some degree pledged to follow up the subject; I therefore send you the subjoined from my note-book, for insertion whenever you may have space for it, in the absence of any more important communication.

*Vomiting.*—On board the Alkmaar hospital-ship in the Baltic, a patient laboured under such constant and violent irritation of the stomach, as to lead us all to believe, at the commencement of the case, that there was considerable morbid action going forward in that organ.

It was the duty of the hospital-mates to be much on the watch, and to notice more particularly such patients as were considered labouring under any acute disease: my attention, therefore, was attracted, among others, to the case under consideration; and observing, for two or three days, that the vomiting only occurred at certain periods,—namely, when the physician to the fleet was paying his morning or evening visit to the sick, we determined the more narrowly to watch this patient, and the result was a complete detection of the imposture. It was effected in the following manner:—

Seeing (as has been already stated) that the vomitings only occurred at stated periods, and that the patient ate his provisions, in the absence of the medical officers, without any vomiting, at Dr. Baird's next visit I watched, and distinctly observed the patient, before the vomiting began, to make violent pressure with his hand, under the bed-clothes, on the region of the stomach; and so convinced was I that the vomiting was entirely produced in this way, that I mentioned the circumstance to the

Doctor, and requested that he would suffer me to visit him a few minutes on the following morning, before he appeared on the hospital deck, that I might secure his hands outside the bed-clothes; and that I would answer for it the patient would not vomit on that occasion, even if he were to administer any medicine to him, however nauseous, short of an emetic.

The arrangement was made: I sat upon the bed, with the patient's hands in mine. The Doctor came at the appointed time, took a chair by me, and exhibited to him an ounce of castor-oil, uncovered by any vehicle, and remained by him one whole hour, without the slightest disposition to vomit evincing itself during the whole period.

In the course of my practice, and in conversation with other medical men since, I have been thoroughly convinced of the existence of this power in certain persons, to excite vomiting, by pressure on the region of the stomach, whenever they pleased. The patient above adverted to was discharged cured to his proper ship, and an account transmitted, to the surgeon, of the imposture practised.

*Disease of the Loins, from Hurts, Falls, or Sprains.*—This is no uncommon source of complaint and imposture among men employed in king's ships and dock-yards, and is very difficult to detect: close observation, watching, and a variety of concurring circumstances, can alone lead to a just conclusion and detection in such cases.

The most remarkable case of this kind that occurred in my practice, was that of a man, named Bradley, who was admitted into Deal Hospital, from a line-of-battle ship in the Downs. He was stated to have fallen from the fore-yard into the sea, striking the region of his loins, in his descent, against the anchor-fluke. A few hours after the accident, I visited this man in the hospital, and, after the most attentive examination of the part pointed out to me by the patient as that which had been so severely injured, I found, to my utter surprise, that there was not the slightest mark of injury, either on the lumbar region or on any other part of his body; but the howling and screaming noise made by the patient at this examination was sufficient, with the circumstances above adverted to, to raise strong doubts in our minds as to the existence of any injury whatever, beyond the alarm occasioned by his fall from the yard into the sea; and which opinion I then distinctly stated to the patient.

Bradley was young, vigorous, and obstinate, and a thorough-bred seaman withal; every attention was therefore paid to him; but for weeks together he declined to take any kind of sustenance, alledging "that it made him sick, and injured his spine, which was broken;" and, as a proof of this, his trunk was bent to nearly a right angle with the lower extremities. He continued, throughout each night, to make such a groaning noise in the ward, from actual pain, as he said, that I was constrained at length, at the earnest request of the other patients, to move him into a small room by himself, and which stood somewhat back from the line of the main building.

When a rug was spread on the floor of his ward, and the patient placed on his back upon it, for the purpose of examination, his legs and thighs were standing upwards at the same angle with his body, as when he walked; and, when his legs were pressed down to the floor, his body started up, and he then appeared in a sitting posture, to the great amusement of the hospital-mates, who assisted me on these occasions. Bradley continued to state, however, that, his back being broken by the villainous anchor, he was no longer fit for the service, and hoped I would invalid him; while the loud and incessant noise he made, even in this distant abode, continued to annoy every body within the sphere of his vociferations.

About this time a companion of his in the lower ward, from whence he had been removed, addressed me one day in a whisper, and said he wished to speak with me alone. This fellow, Hopkins, had just had his own leg emancipated from the wooden boot, and, by way of making himself useful to me, with the view that he might not himself be sent off to his ship with a branded ticket, (of which seamen stand in great terror,) he addressed me as follows:—"You are quite right, sir, with respect to Bradley: he is a great rogue, and pretends not to be able to eat or to stand erect; but I have watched him,—have seen him get out of bed in the middle of the night, when he thought we were all asleep, and walk to the cupboard at the end of the ward as uprightly as you can walk, and where he uniformly every night made a hearty repast from the left provisions. He would then return to his bed in like manner, observing the utmost care and quiet, lest his comrades should be disturbed, and detect him." Hopkins further stated, that Bradley had sent for him that very morning, when the latter bade him good bye, if he would not accompany him; and, in confidence, told him that, as the hospital-clock struck eleven that night, he would make his escape in a certain direction, across the green, and over the burial-ground wall; but at the same time strenuously urged the latter to join him in his escape from the lower ward.

Upon this information, I directed the serjeant of the guard, after dark, where the soldiers should be planted to secure the deserter; placing myself in the operation-room, with all the hospital-mates, and the nurse of Bradley's ward; when, sure enough, just as the clock struck eleven, it was announced that there were white sheets thrown down from the window of Bradley's room on the first floor, by which he descended very gently and safely. He then ran across the back green to the wall mentioned by Hopkins, with all the uprightness and agility of a young man free from disease or injury of any kind; and, on the following morning, when I visited him in the guard-room, he was very penitent, but appeared, when standing, to be still bent double. On his asking me to show him mercy, and not report him for punishment, I told him that, if he expected mercy at my hands, he would that instant stand erect as he was when I saw him run across the green the previous night. He complied, stood up like a soldier on parade, and was discharged to his duty on-board his ship.

In this very dock-yard from which I write, a man complained of having sprained his loins in the execution of his duty. He was put on the hurt list, which entitles the individual to receive half his full pay; and there he continued a considerable time. He was declared not to be a fit object to receive the bounty of government for his reported injury, and he was discharged the yard without a pension. On the following day, this very man, who for weeks before had been bent double, and stated himself to be perfectly unable to work, was recognised by an officer, now here, at the top of a long ladder in the town, with a brick-layer's hod upon his shoulder, and, on descending, he was observed to walk away perfectly upright.

*Epilepsy.*—This is another disease not unfrequently simulated among seamen and soldiers, and sometimes even in domestic life.

The state of the pupil will, in most instances, lead to a discovery of this fact,—namely, as to the influence of light upon the iris, occasioning a contraction or dilatation.

I have, however, had brought to my recollection, by my friend and former messmate, Dr. Burnet, late assistant physician to Greenwich Hospital, the case of a man on-board the Isis, fifty-gun ship, on our passage to Newfoundland in 1802, who had epileptic fits very frequently, and to which he said he had been subject for some years. His pupils, during the paroxysm, were uniformly natural when light was opposed to them; we were, therefore, satisfied that the disease was feigned. But, to put the patient to another test, I introduced some fine dry Scotch snuff into a quill, and, while the patient was in one of his fits, the snuff was blown up his nostrils, which induced another fit—a fit of sneezing, that lasted nearly a quarter of an hour.

I have since tried snuff in real cases of epilepsy, and never once could produce that effect, although the individuals were not snuff-takers. This, then, is a simple and gentle mode of detection, to which there can be no possible objection. The person in the Isis never had another fit, while Dr. Burnet and I remained in the ship.

During the first year or two that the Penitentiary at Millbank was in operation, Jane Malcolm, a female convict, feigned epilepsy; and, upon being convinced of the fact, I directed the resident apothecary to dissolve some aloes and salts in a pint of water, and which I administered to her myself on one occasion, when she was labouring under a very severe paroxysm of this disease. The bitter and nauseous taste of the medicine made her resist taking it with vehemence, and, in the struggle, she bit the tin vessel which contained it, and broke a rotten tooth; of which she afterwards complained, to the visitor, as very cruel and unkind treatment received at my hands.

The above remedy, however, cured the disease; for she never had another fit while I continued attached to the institution, which was some years.

*Œdematous Swellings of the Extremities, &c.*—It occasionally occurs that arms and legs in this state are presented to the notice of medical officers, not only in the navy and army, but to those of prisons and prison-ships also; and I have a perfect recollection of such cases having occurred in my own practice many times, in the hospital and elsewhere. This swelling is produced by the tight application of a ligature, higher on the limb than is thought proper to exhibit to the view of the surgeon. The mode of detection is here obvious enough, and need not, therefore, be more adverted to in this place; but, perhaps, the most complete mode of detection would be to visit the patient, and examine the part while the ligature is actually applied and in operation, which is generally from a quarter of an hour to an hour and a half, or two hours, earlier than the customary visit of the medical officer.

When detection has taken place in this way, I have more than once been replied to by the person, with more ingenuity than truth, that the ligature was applied for the purpose of allaying pain. In one case, indeed, I felt considerable embarrassment; for, when the mark of the ligature had been distinctly witnessed by me two or three different times, yet the arm continued to be swelled afterwards, certainly without its use, for there was no mark: when at length we discovered that it was produced by his hanging his arm over the back of a chair, and making considerable pressure upon the course of the axillary vessels, for some time previous to our customary visit.

*Hæmoptysis, Hæmatemesis, or Spitting of Blood, &c.*—being a communication from a friend.

"As several devices are resorted to by persons belonging to the public service to obtain pensions undeservedly, I beg to relate a few of the many attempts practised for that purpose, which have come under my observation.

"A robust, powerful man, of middle age, stated himself unable to work, from an injury of the slightest description, and resolutely persevered in denying himself the common means of sustenance, by which, with close confinement to his miserable lodging, and keeping constantly in bed, some degree of debility and fever was induced.

"When he was tired of this mode of punishment to gain his ends, he endeavoured to work upon the minds of the officers, by creeping into their presence, claiming their compassion, and sometimes enforcing the representations of his sufferings by disgorging small quantities of blood from his mouth, (which it was well ascertained he had procured at a butcher's shop in the town,) feigning a hectic cough at the time, and thus exhibiting symptoms which were thought infallible. Even this expedient failed, and at length, finding all his ingenuity of no avail, he thought proper to regain his usual health when discharged without a pension.

"Another character, with similar views and pretensions, while on night-watch, quietly walked into one of the docks, placed himself in an immovable position, bellowing out for help, as he had fallen from the brow of the dock, and was almost killed. Immediate notice was taken of this supposed accident, and, on inspection, the total absence of all external marks of injury, together with regular pulsation, made his case very suspicious. However, after using all means of advice, admonition, and remonstrance, assisted by the frequent use of blisters and medicine of no very palatable cast, he still carried on the farce, with the hope of ultimately succeeding, and, by privations of all sorts, reduced himself to a mere shadow.

"Finding his friend's scheme of spitting blood fail him, a paralysis of his lower extremities kept up the deception a little longer; and, when placed on the discharge-list, he took his departure without the much wished-for pension, and very shortly recovered the use of his limbs."

In my own practice, both public and private, I have known instances of hæmoptysis feigned by cutting the gums, or roof of the mouth, with a penknife, or other sharp instrument. The incisions being visible, no doubt could be entertained of the reality of the imposture; and a worthy colleague of mine, at the Westminster General Dispensary, has mentioned to me the case of a young lady, a private patient of his, who feigned hæmoptysis by mixing with her saliva some vermilion paint.

*Pains, Spasms, and Disease of the Stomach and Bowels.*—In a convict hulk at this port, entirely for the reception of boys, (who are either employed as shoemakers, tailors, or book-binders, and some of whom recognised me as an old Penitentiary acquaintance,) several instances of feigned disease have occurred: but here I wish more particularly to advert to the class of shoemakers, several of whom, to avoid work, and be sent on-board the hospital-ship, produced actual and very serious mischief to the stomach and bowels, by swallowing quantities of the sulphate of iron (copperas), used in their trade.

One of these cases was shown to me by the intelligent surgeon of the hulk, Mr. A. Robertson; and who has informed me since that this boy is dangerously ill, and likely to die, in consequence of such malpractice.

From the foregoing observations, it may be supposed, and naturally enough, that I may have now and then erred in my judgment, and occasionally accused an innocent man of imposture. I now take this opportunity to declare, on my honour, (and there are many living witnesses to bear me out in the assertion,) that it has been my good fortune never once to have accused an individual of being an impostor, or even to treat him as such, who was not eventually proved to be one. The candid reader will, I am sure, admit that I owe to myself this justification, after the melancholy catalogue of impostures which have been detailed in your Journal from my pen.

*Royal Dock-yard, Sheerness; June* 1825.

*** We are tempted to add to the above remarks of our esteemed correspondent, a case of simulated disease, which occurred several years ago in the first regiment of Guards, chiefly on account of the very ingenious manner in which it was overcome by the late surgeon-major of the regiment, Mr. Charlton. A soldier had been long in the hospital, in consequence of having lost, as he asserted, the use of his right arm; nor could the motions of the limb be restored by any means whatever. There were the strongest reasons for believing that the complaint was wholly fictitious. At length Mr. Charlton suggested the following plan:—The man was prevailed upon one evening to suffer the *sound* arm to be fastened securely to the side, with the hope, as he was told, of gradually restoring the motions of the *diseased limb*, by making him entirely dependent upon its use. In this condition the man went to bed as usual; and, in the middle of the night, the orderly man was directed, in the presence of the resident assistant-surgeon, and some of the patient's comrades, to irritate the nostrils with a feather: this produced an instantaneous motion of the diseased arm, which was applied with great energy to the part tickled. The man was then awoke, and congratulated by the spectators on having thus suddenly recovered the use of his limb. The cure was complete and permanent.—Editors.

